# Frequent Arousals from Winter Torpor in Rafinesque’s Big-Eared Bat (*Corynorhinus rafinesquii*)

**DOI:** 10.1371/journal.pone.0049754

**Published:** 2012-11-21

**Authors:** Joseph S. Johnson, Michael J. Lacki, Steven C. Thomas, John F. Grider

**Affiliations:** 1 Department of Forestry, University of Kentucky, Lexington, Kentucky, United States of America; 2 United States National Park Service, Mammoth Cave, Kentucky, United States of America; 3 Department of Biology, University of North Carolina, Greensboro, North Carolina, United States of America; University of Regina, Canada

## Abstract

Extensive use of torpor is a common winter survival strategy among bats; however, data comparing various torpor behaviors among species are scarce. Winter torpor behaviors are likely to vary among species with different physiologies and species inhabiting different regional climates. Understanding these differences may be important in identifying differing susceptibilities of species to white-nose syndrome (WNS) in North America. We fitted 24 Rafinesque’s big-eared bats (*Corynorhinus rafinesquii*) with temperature-sensitive radio-transmitters, and monitored 128 PIT-tagged big-eared bats, during the winter months of 2010 to 2012. We tested the hypothesis that Rafinesque’s big-eared bats use torpor less often than values reported for other North American cave-hibernators. Additionally, we tested the hypothesis that Rafinesque’s big-eared bats arouse on winter nights more suitable for nocturnal foraging. Radio-tagged bats used short (2.4 d ± 0.3 (SE)), shallow (13.9°C ± 0.6) torpor bouts and switched roosts every 4.1 d ± 0.6. Probability of arousal from torpor increased linearly with ambient temperature at sunset (*P*<0.0001), and 83% (*n* = 86) of arousals occurred within 1 hr of sunset. Activity of PIT-tagged bats at an artificial maternity/hibernaculum roost between November and March was positively correlated with ambient temperature at sunset (*P*<0.0001), with males more active at the roost than females. These data show Rafinesque’s big-eared bat is a shallow hibernator and is relatively active during winter. We hypothesize that winter activity patterns provide *Corynorhinus* species with an ecological and physiological defense against the fungus causing WNS, and that these bats may be better suited to withstand fungal infection than other cave-hibernating bat species in eastern North America.

## Introduction

Many bat species inhabiting temperate climates rely heavily on torpor during winter to survive extensive periods of cold and reduced food availability [1]. Winter torpor, or hibernation, typically consists of numerous discrete torpor bouts separated by brief periods of normothermy, or arousal, which are proposed to serve numerous physiological purposes [2], [3], [4], [5]. Laboratory and field studies of winter torpor show that frequency of periodic arousals varies among species, as do minimum torpid body temperatures (*T*_b_) [1], [6], [7], [8]. Together, depth and duration of torpor bouts are part of an energy conservation strategy in which successful hibernators balance the costs and benefits of arousals with costs and benefits of torpor [9], [10], [11]. Although data from field studies are scarce, it is likely that winter torpor strategies vary within and among species, with animals possessing higher body mass and lower surface area to volume ratios able to survive more frequent arousals, longer periods of normothermy, or higher torpid body temperatures [9], [12]. Furthermore, regional climates and the unique ecology and life history of each species are also likely to impact seasonal torpor strategies.

Understanding differences in winter torpor behaviors among bat species is of heightened interest due to fatal disruptions in hibernation resulting from white-nose syndrome (WNS). The causal agent of WNS is a cold-tolerant fungus, *Geomyces destructans*, which is believed to be of Old World origin and only recently introduced to North America [13], [14], [15], [16]. Many bats presumed to have died from WNS have little to no fat reserves remaining, leading to the hypothesis that fungal infection causes more frequent and/or longer duration arousals during hibernation [7], [16], [17], [18]. Ultimate and proximate causes of mortality in infected bats are likely to be more synergistic, however. For example, erosion of the skin from fungal invasion may negatively affect water balance, resulting in dehydration, increased frequency of periodic arousals, and additional complications with thermoregulation [19], [20].

Neither the clinical signs of WNS or the presence of *G. destructans* have been observed in bats in the genus *Corynorhinus* (big-eared bats). Although expansion of the fungus into the range of *Corynorhinus* species is currently limited, WNS has been documented in five caves used by the endangered Virginia big-eared bat (*Corynorhinus townsendii virginianus*), including the largest known hibernaculum of the species, without visibly affecting any big-eared bat [21]. Currently, experimental data showing *Corynorhinus* species to be less vulnerable to WNS are lacking, and it is unknown whether one or more aspects of the ecology or physiology of these bats could provide a defense against fungal infection.

Rafinesque’s big-eared bat (*Corynorhinus rafinesquii*) is a small (8–14 g) forest-dwelling bat found throughout the southeastern United States [22], [23]. Rafinesque’s big-eared bat hibernates in caves and mines in mountainous and karst portions of the species range, but these hibernacula are largely absent in southern portion of the range, where big-eared bats have been documented hibernating in trees, wells, and cisterns [23], [24], [25]. While there are no data on the depth or duration of winter torpor bouts in Rafinesque’s big-eared bat, observations of winter mating and bats frequently moving among hibernacula suggest this species may undergo torpor bouts of shorter duration than other cave-hibernating bat species in eastern North America, making it an ideal species for winter studies [22], [26], [27].

Herein we evaluate winter torpor behaviors in Rafinesque’s big-eared bat. We hypothesized that Rafinesque’s big-eared bats use less torpor than other cave-hibernating bat species in eastern North America, predicting that Rafinesque’s big-eared bats use shallower, shorter duration torpor bouts than those observed in other eastern North American species. Additionally, we hypothesized that Rafinesque’s big-eared bats are active foragers throughout the winter, predicting that arousals would be associated with sunset, and would be more likely to occur on warmer nights.

## Materials and Methods

### Study Area

Field work occurred at Mammoth Cave National Park (MCNP; 37.2072° N, 86.1319° W) in Barren, Edmonson, and Hart counties, Kentucky, USA. The area is predominantly forested and is dissected by numerous small drainages, creating a topographically diverse landscape. Forest cover consists of oak-hickory (*Quercus* − *Carya* spp.) and western mixed mesophytic forests [28]. During summer, Rafinesque’s big-eared bat roosts in hollow trees, sandstone outcrops, caves and abandoned man-made structures (J Johnson, unpublished data). Hundreds of caves occur within the 21,380 ha Park, including six known hibernacula of Rafinesque’s big-eared bat. The Park has one of the largest known winter concentrations of Rafinesque’s big-eared bats, with >1000 big-eared bats hibernating within the Park [23].

### Radio-telemetry Data Collection and Analysis

All methods were approved by the University of Kentucky Institutional Animal Care and Use Committee (IACUC No. A3336-01) and the National Park Service (NPS IACUC No. 2011-30). We captured Rafinesque’s big-eared bats hibernating in caves and abandoned buildings during three consecutive winters and radio-tagged bats during March 2010, January 2011, and November 2011–January 2012. We combined data across winters and divided the dataset into bats radio-tracked during early- (mid-November–early December), mid- (mid-December–mid February), and late-winter (mid-March–early April) to account for variability in torpor bout duration associated with progression of the hibernation season [29], [30], [31]. No radio-tagged bat was tracked during >1 winter period.

We recorded age, sex, reproductive condition, right forearm length, and body mass of captured bats. We determined the body condition of all but 4 bats (forearm lengths were not measured for 4 individuals) by dividing body mass by forearm length [32]. We compared body condition between sexes and among winter periods using a two-way analysis of variance (ANOVA). We used a 0.05 significance level for difference and compared least squares means using Tukey’s adjustment when significant, for all ANOVAs. We tested all datasets for homogeneity of variance using Variance Ratio F_max_-tests.

A subset of bats were fitted with 0.52 g temperature-sensitive radio-transmitters (model LB-2T, Holohil Systems, Ltd., Carp, Ontario) immediately below the shoulder blades using surgical adhesive (Perma-Type, Plainville, CT) [33]. We placed radio-transmitters below the shoulder blades to avoid concentrations of brown adipose tissue which may lead to errors in estimation of *T*_b_ during periods of non-shivering thermogenesis [34]. Bats were placed back in their roosts after the adhesive was allowed to dry (ca. 15 min), by which time they had aroused from torpor. We deployed HOBO dataloggers (models U23-001 or UA-002-08, Onset Computer Corporation, Bourne, MA) inside and outside roosts after releasing radio-tagged bats. Dataloggers recorded air temperatures inside roosts (*T*_r_) and outside roosts (hereafter ambient temperature, *T*_a_) at 15-min intervals. Dataloggers recording ambient temperatures were placed inside solar radiation shields.

Each radio-transmitter was individually calibrated by the manufacturer, providing a unique polynomial equation for use in converting transmitter pulse rate into skin-temperature (*T*_sk_). *T*_sk_ of each radio-tagged bat were recorded by three datalogging receivers (model R4500S, Advanced Telemetry Systems, Inc., Isanti, MN) placed in watertight boxes with an external power source. Receivers were programmed to scan for radio-tagged bats at 5-min intervals and placed outside caves and abandoned buildings. Receivers were checked weekly or bi-weekly for maintenance and moved to new locations when necessary. We attempted to locate bats in nearby caves and buildings if their radio-signals were not heard outside monitored hibernacula. Chronological accounts of the roost location of each bat were determined based upon which receiver recorded daytime signals. Only two roosts (buildings) were located close enough to each other that bats in either roost could be recorded by a single receiver. Recorded signal strength differed notably between these roosting sites, however, allowing for a clear determination of roosting location. Bats which could not be located for several days (always ≤10 d) were considered to be in the same roost for the entire period. We compared roost-switching frequencies (i.e., number of days a bat inhabited a roost before switching to a new roost) between sexes and among winter periods using a two-way ANOVA.

We applied Willis’ [35] equation for an energy-based temperature threshold for torpor onset (*T*_onset_), using the conservative equation based upon model parameters minus 1 SE. This equation requires a simultaneous measure of *T*_r_, and we were only able to calculate *T*_onset_ when bats occupied roosts with HOBO dataloggers. Calculated values for *T*_onset_ varied marginally (between 31.5−32.3°C), however, and we applied a *T*_onset_ value of 32°C to all bats. Thus, we considered bats to be torpid when *T*_sk_ was <32°C, and considered torpor bouts over when *T*_sk_ >32°C or if radio-signals were lost following a rapid rise in *T*_sk_; presumably signifying the bats left the roost before normothermic *T*_sk_ was recorded.

Although Willis’ [35] equation was designed for use with *T*_b_, not *T*_sk_, it still holds advantages over an arbitrary definition of torpor because *T*_sk_ in bats is a good indicator of *T*_b_ [36]. Because *T*_sk_ is typically a few degrees below *T*_b_, especially at low *T*_a_, use of the *T*_onset_ equation likely results in a *T*_onset_ slightly below that which would be obtained using *T*_b_ measurements. However, error in determining when bats in our study entered torpor resulting from use of *T*_sk_ is likely on the scale of several minutes because we did not observe *T*_sk_ lingering between 32°C and other typical torpor cutoffs, such as 25°C [37]. Instead, *T*_sk_ quickly dropped to values <20°C while bats roosted in hibernacula. Error in determining arousal from torpor may be greater because of the concentration of brown adipose tissue located beneath the skin, potentially leading to erroneous measures of *T*_sk_. While our data are insufficient to determine the extent of this error, we contend that it is likely small, because initiation of rapid heat production through non-shivering thermogenesis likely signifies that the bat is either undergoing spontaneous arousal or maintenance of a *T*_b_ notably higher than *T*_r_. In the former scenario, error in determining the length of torpor bouts is likely to be a small period of time. In the latter scenario, torpid *T*_sk_ should not exceed *T*_onset_.

Bats were considered torpid for the entire time they were not recorded by any datalogging receiver (always ≤10 d). While this might overestimate torpor bout duration while un-located (bats may have aroused and not left the hibernaculum during this time), it provides a conservative measure for testing the prediction that Rafinesque’s big-eared bats use shorter duration torpor bouts compared to other species. We determined the duration (days), min *T*_sk_, and average *T*_sk_ of each torpor bout, and averaged each measure within bats for statistical analysis. We also determined the average duration (hrs) of normothermic periods when entry and arousal from torpor were successfully recorded for two consecutive torpor bouts. Each variable was compared between sexes and among winter periods using a two-way ANOVA.

We determined the time difference (hrs) between arousal and sunset on days where we recorded torpid *T*_sk_ data prior to arousal. We used generalized estimating equations (PROC GENMOD, SAS) to assess the role of varying ambient temperatures on the probability of arousal from torpor [38]. Generalized estimating equations are an extension of the general linear model, but do not provide correlation coefficients. The strength of generalized estimating equations is that they allow for analysis of clustered (i.e., many days of observation from an individual bat which are not independent samples) binary (i.e., torpid or active) data which cannot be fitted using typical linear models.

We calculated the Heterothermy Index (HI) for each bat to quantify variability in *T*_sk_ of each individual [39]. We followed the recommendation of Boyles et al. [39] to use the mode representing the greatest *T*_b_ as *T*_b-opt_. We used all of the recorded *T*_sk_ for individual bats when determining each HI value.

### PIT-tagging

We used a harp-trap (Faunatech, Bairnsdale, Victoria, Australia) to capture Rafinesque’s big-eared bats exiting a man-made roost during April and August 2011. We measured each bat as described above, and subcutaneously implanted a 12.5 mm PIT tag (model TX1411SST, Biomark, Inc., Boise, ID), sealing the injection site with surgical adhesive (Perma-Type, Plainville, CT), as part of a long-term study overseen by MCNP’s Science and Resources Management Division. Roost entrances were surrounded by custom-made antennas designed to read and record PIT tags of marked bats entering or exiting the roost. We summarized PIT tag readings into the number and identity of tagged bats passing through the sensor field each day between 03 May 2011 and 01 April 2012. Because bats were often recorded at the roost more than an hour after sunrise and an hour prior to sunset, we considered a day to be the 24 hr period between 1200 and 1200 the following day. This allowed us to efficiently process large quantities of data while ensuring we captured all nightly activity in our summaries. We report the percentage of adult males, adult females, male young-of-the-year, and female young-of-the-year recorded each day.

We performed a linear regression (PROC REG, SAS) to evaluate the role of ambient temperatures on winter activity, and test our prediction that bats would be more active on nights suitable for nocturnal foraging. We used number of marked bats recorded each day during winter as the response variable, *T*_a_ at sunset as the independent variable, and a significance level of 0.05. Winter was defined as 01 November–01 March. We did not include data from March 2012 in this analysis due to unusually high temperatures and high activity of bats at the roost (see Results). A separate analysis was conducted for adult males, adult females, male young-of-the-year, and female young-of-the-year.

## Results

### Radio-telemetry

We captured and measured 33 bats during periodic roost searches. Body condition differed among bats (*F*_3, 29_ = 60.9, *P*<0.0001), with differences detected among bats captured during different winter periods (*F* = 90.9, *P*<0.0001), but not between sexes (*F* = 0.39, *P* = 0.54). Mean body condition was greatest during early-winter (0.28±0.01) compared to mid- (0.21±.001, *P*<0.0001) and late-winter (0.19±0.001, *P*<0.0001), and mean body condition was greater during mid-winter than late-winter (*P* = 0.006). Body conditions of males and females both averaged 0.23±0.01. Body mass of females averaged 10.0±0.46, versus 10.0±0.52 for males. Range in body mass declined from 11.4–14.0 g (body conditions: 0.27–0.32) in mid-November to 7.1–8.3 g (body conditions: 0.16–0.20) in early March.

We radio-tagged 14 female and 10 male Rafinesque’s big-eared bats between 2010 and 2012. All bats re-entered torpor following radio-tagging but aroused from torpor within 1 hr of sunset. These initial bouts were not included in analyses or summaries. Four females were never located following emergence from hibernacula on the first night and were not included in any torpor analyses or summaries. Bats switched roosts every 4.1 d ±0.6, with no difference detected between sexes or among winter periods (*F*_3, 16_ = 0.88, *P* = 0.47). Bats traveled 2535±437 m (range = 549–5964) between consecutive roosts. We were able to determine HI values for 8 males and 8 females. Optimal skin temperatures ranged from 28–34°C (mean = 32.8±0.4) and HI ranged from 14.4–20.7 (mean = 17.7±0.5).

**Figure 1:**
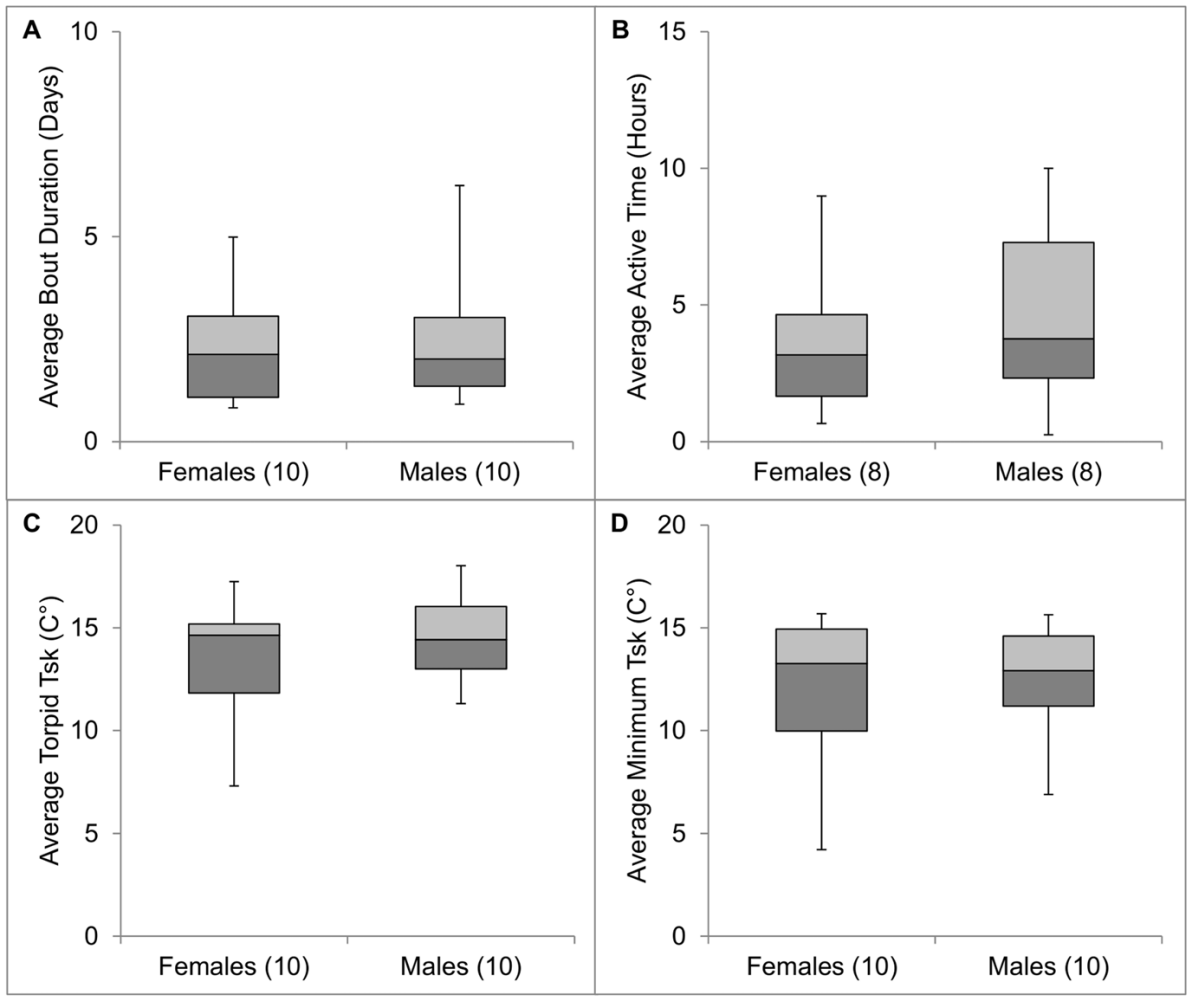
Winter torpor behaviors of male and female Rafinesque’s big-eared bats determined through radio-telemetry at Mammoth Cave National Park, Kentucky, USA. Number of radio-tagged bats is included in parentheses.

**Figure 2:**
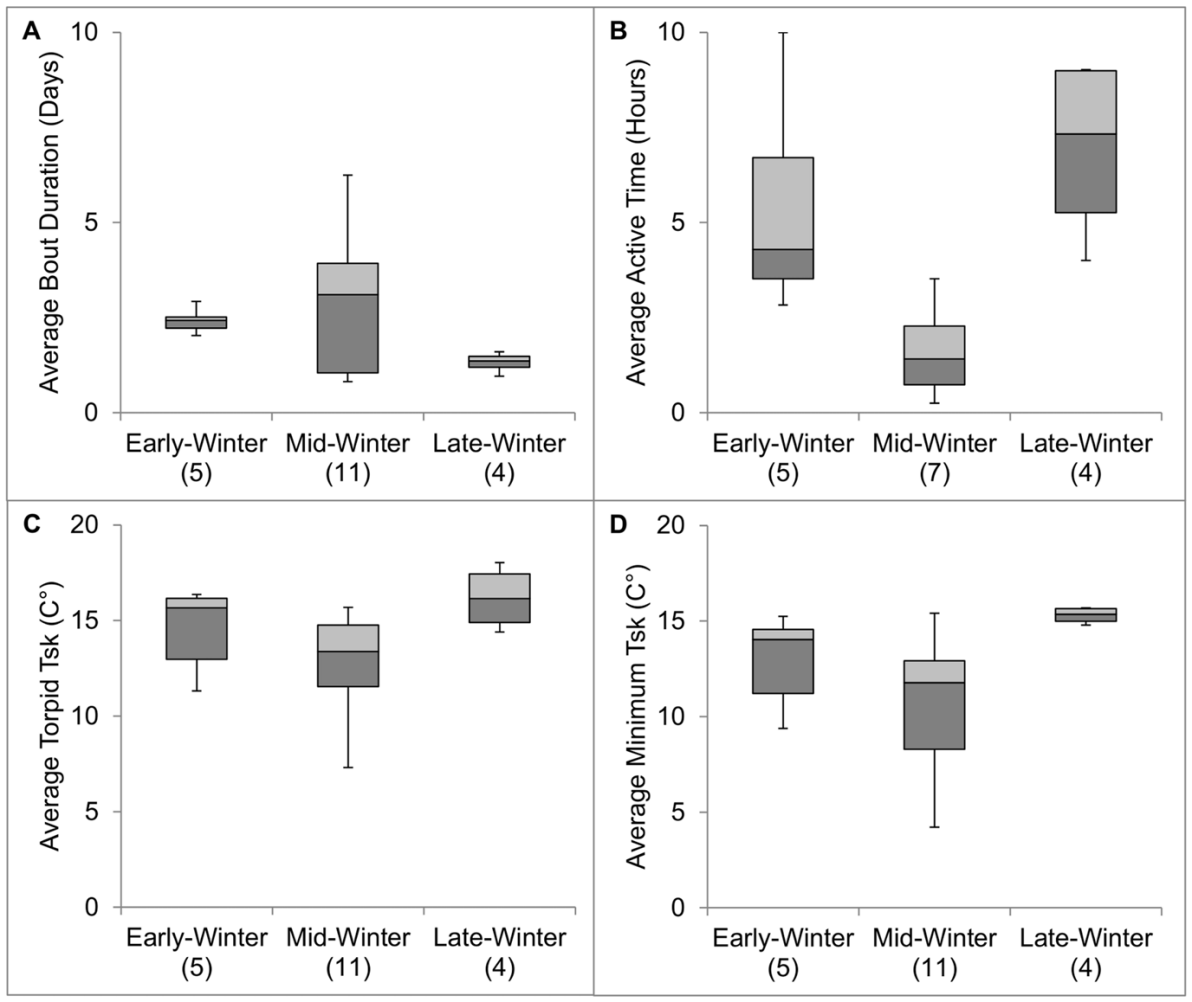
Winter torpor behaviors of Rafinesque’s big-eared bats during early (mid-November–mid-December), mid- (mid-December–mid-February), and late (March) winter determined through radio-telemetry at Mammoth Cave National Park, Kentucky, USA. Number of radio-tagged bats is included in parentheses.

We documented 7.4±1.2 (range = 1–16, *n* = 147) torpor bouts per bat. Bats aroused from torpor every 2.4 d ±0.3 (Fig. 1A, 2A), with no difference detected between sexes or among winter periods (*F*_3, 16_ = 0.94, *P* = 0.44). Duration of normothermic periods between torpor bouts differed among bats (*F*_3, 12_ = 5.72, *P* = 0.011), with differences detected among winter periods (*F* = 8.27, *P* = 0.006, Fig. 2B), but not between sexes (*F* = 0.01, *P* = 0.91, Fig. 1B). Duration of normothermy was shorter during mid-winter than early (*P* = 0.039) and late winter (*P* = 0.008). Only one torpor bout was successfully monitored for four bats radio-tagged during January 2011, and duration of normothermic periods were not determined for these bats. Skin temperatures infrequently fell below 10°C during torpor. Average torpid *T*_sk_ was 13.9°C ±0.6 (Fig. 1C, 2C), with no difference detected between sexes or among winter periods (*F*_3, 16_ = 1.74, *P* = 0.20). Minimum torpid *T*_sk_ averaged 12.1°C ±0.8 (Fig. 1D, 2D), with no difference detected between sexes or among winter periods (*F*_3, 16_ = 1.87, *P* = 0.18).

**Figure 3:**
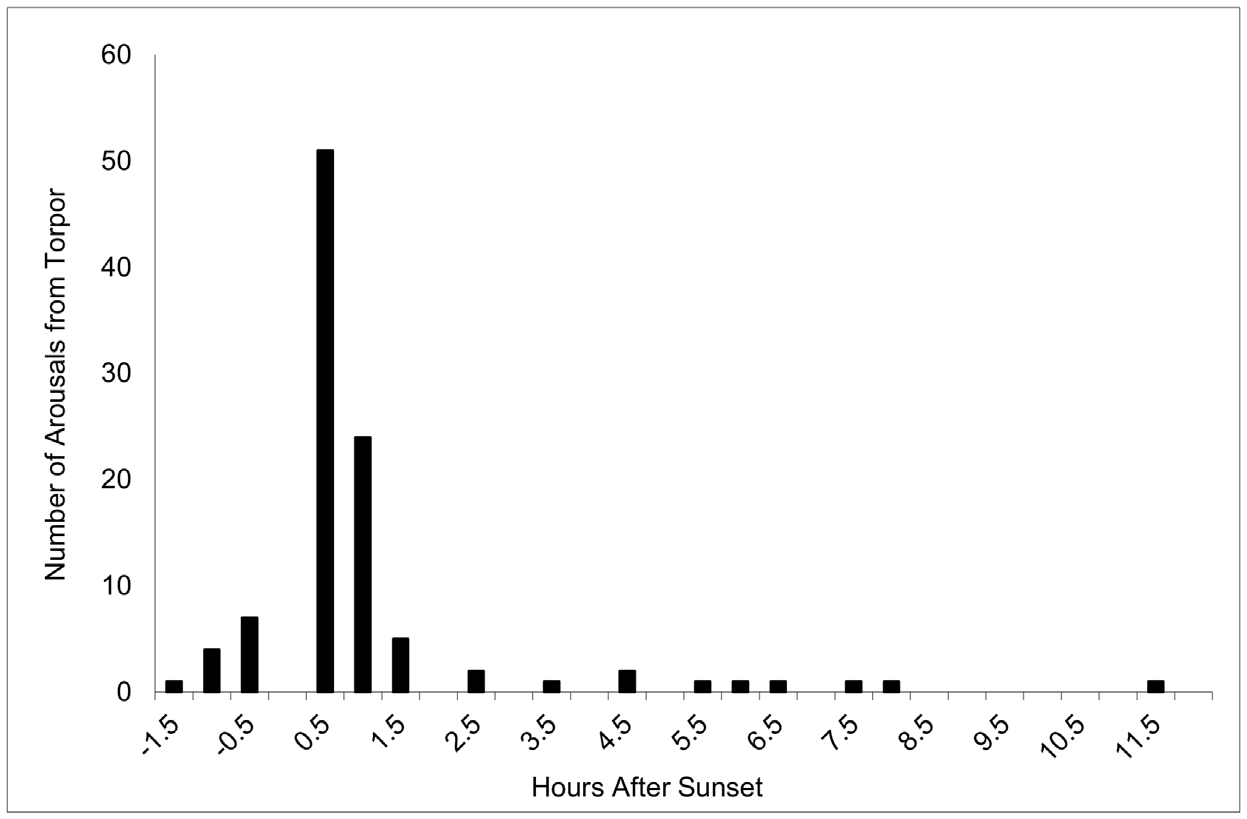
Timing of periodic arousals from hibernation in relation to sunset determined through radio-telemetry at Mammoth Cave National Park, Kentucky, USA.

**Figure 4:**
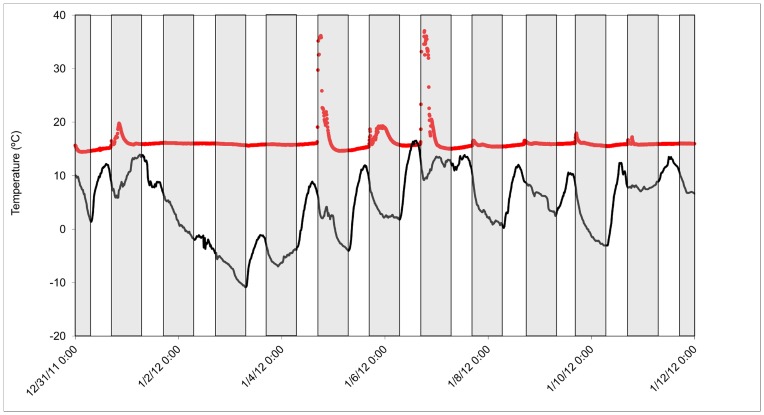
Skin temperatures (red circles) of a female Rafinesque’s big-eared collected through radio-telemetry and concurrent ambient temperatures outside the cave (black line) recorded over 12 days at Mammoth Cave National Park, Kentucky, USA. Shaded areas include hours between sunset and sunrise.

Increasing ambient temperature at sunset significantly increased the probability of arousal (β = 0.13±0.03, *P*<0.0001), and the timing of arousals was centered on sunset, with 50% (*n* = 51) occurring in the 30 min following sunset, and 83% (*n* = 86) occurring within ±1 hr of sunset (Fig. 3). The majority of arousals were documented while bats were hibernating in caves (*n* = 84, 82%), where *T*_r_’s were between 5° and 11°C throughout the winter. Many bats hibernating in caves exhibited periods of rapid thermogenesis occurring within 1 hr of sunset that were not considered arousals, because *T*_sk_ failed to reach 20°C before declining (Fig. 4). Temperatures inside buildings, where 18% (*n* = 19) of arousals were documented, ranged from −8 to 21°C.

### PIT-tagging

**Figure 5:**
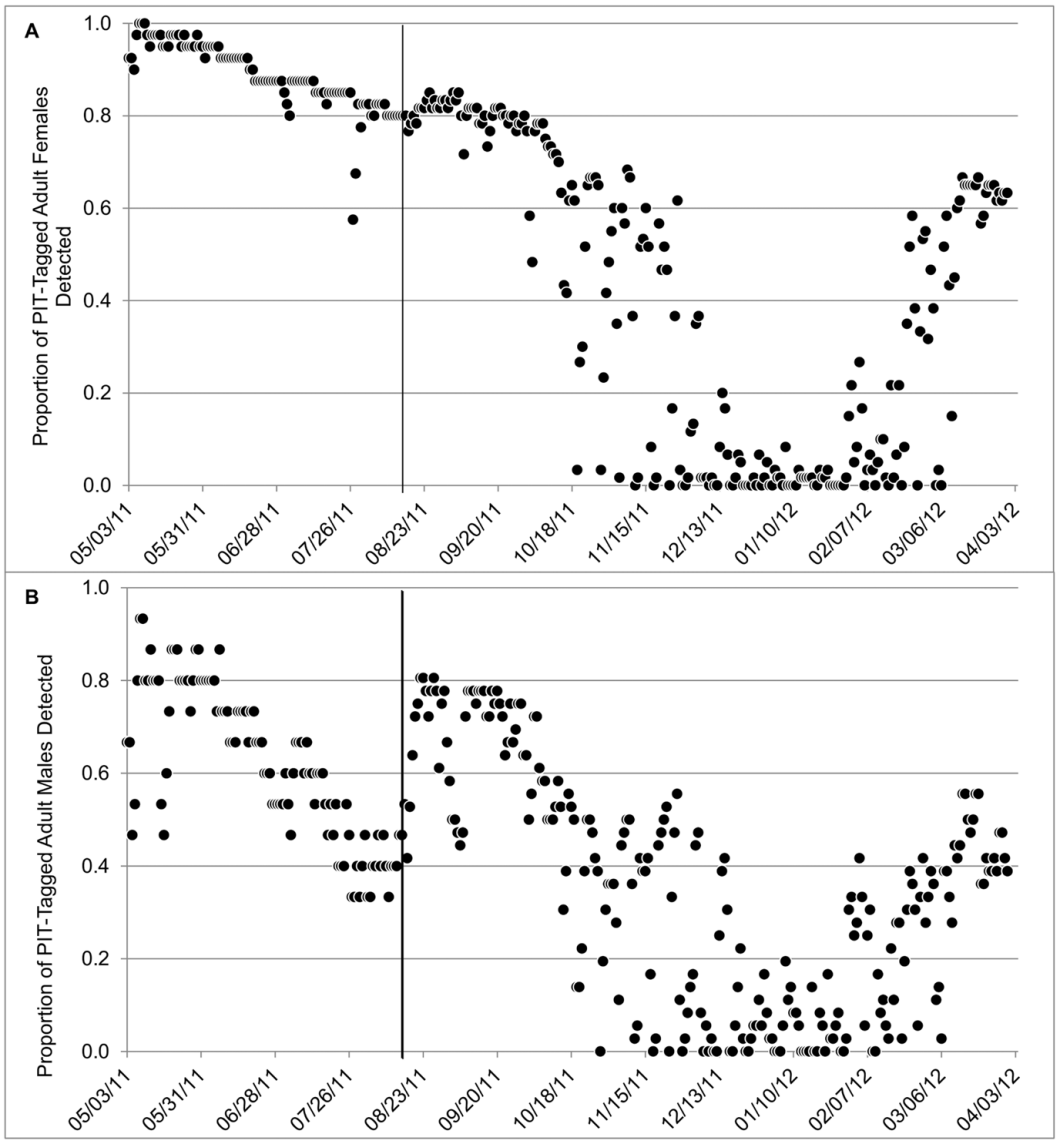
Daily activity of PIT-tagged adult female (A) and male (B) Rafinesque’s big-eared bats at a man-made structure in Mammoth Cave National Park, Kentucky, USA. Solid line indicates a second PIT-tagging effort in mid-August, increasing the number of PIT-tagged bats.

**Figure 6:**
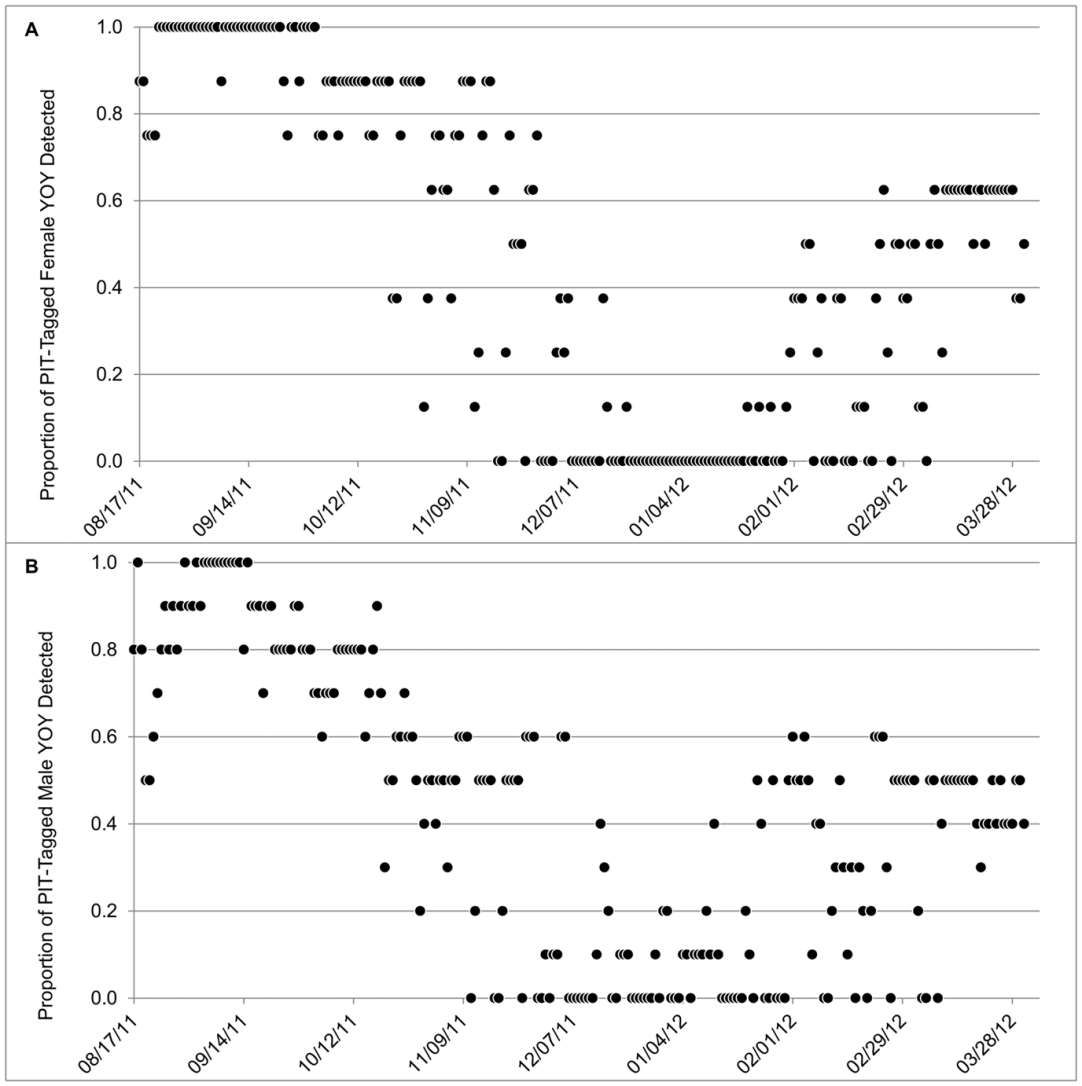
Daily activity of PIT-tagged female (A) and male (B) Rafinesque’s big-eared bat young-of-the-year at a man-made structure in Mammoth Cave National Park, Kentucky, USA.

We PIT tagged 128 Rafinesque’s big-eared bats (38 adult males, 71 adult females, 10 juvenile males, and 9 juvenile females). PIT-tags from 13 bats (11 adult females and 2 adult males) were found at the base of the roost before the end of the study, either as a result of mortality or being shed, and these bats were removed from our analysis. Daily activity of adult females declined in early October, and ≤20% of all adult females were recorded on all days between 04 December and 01 February (Fig. 5A). Fewer than 10% of PIT-tagged adult females were detected on 89% of days between 01 December and 01 February (*n* = 55), and no adult female was detected on 40% of days (*n* = 25). Daily activity of adult females began to resemble fall activity patterns by early March. Number of adult females detected per day during winter increased linearly with *T*_a_ at sunset (*r* ^2^ = 0.23, *F*_1,119_ = 35.1, *P*<0.0001). Activity of female young-of-the year was similar to that of adult females (Fig. 5A, Fig. 6A), and also increased linearly with *T*_a_ at sunset (*r* ^2^ = 0.27, *F*_1,119_ = 44.7, *P*<0.0001).

Daily activity of adult males declined steadily throughout the summer, but remained relatively high during the winter compared to females (Fig. 5B). More than 20% of PIT-tagged males were detected on 13% of days between 01 December and 01 February (*n* = 8), while fewer than 10% of males were detected on 71% days (*n* = 44), and no male was detected on 32% of days (*n* = 20). Number of adult males detected per day during winter increased linearly with *T*_a_ at sunset (*r* ^2^ = 0.26, *F*_1,119_ = 41.8, *P*<0.0001). Activity of male young-of-the year was similar to that of adult males (Fig. 5B, Fig. 6B) and also increased linearly with *T*_a_ at sunset (*r* ^2^ = 0.19, *F*_1,119_ = 28.1, *P*<0.0001).

## Discussion

Rafinesque’s big-eared bats hibernating in caves and abandoned buildings used short, shallow torpor bouts, and bats were frequently active during winter. Torpor patterns did not differ between male and female big-eared bats, but consistently varied with the progression of winter. Normothermic periods were significantly shorter during mid-winter, and while torpor bout duration and *T*_sk_ did not differ significantly among winter periods, we recorded the longest torpor bouts and lowest *T*_sk_’s during mid-winter. These findings are consistent with other mammalian hibernators, where torpor bouts are longest, and normothermic periods shortest, during the middle of the hibernation season [29], [30], [31]. Probability of arousals increased with increasing aboveground *T*_a_ at sunset, and it is likely that the numerous warm days occurring during the mid-winter of 2011–2012, when 50% of days (*n* = 29) had daily high temperatures exceeding 10°C, were partly responsible for the large variance in torpor duration among bats. Thus, while the longest torpor bouts occurred during mid-winter, short torpor bouts were also common during this period, resulting in substantial variation and lack of differences in bout duration among winter periods.

Similar patterns in torpor use and body conditions between males and females throughout the winter are not consistent with the thrifty female hypothesis [10], [37]. The thrifty female hypothesis predicts that females are more conservative with fat reserves during winter because it is critically important to carry some of these reserves into spring, when gestation begins. To accomplish this, females use longer torpor bouts at lower temperatures than males. Males are not as energetically constrained in spring, and, therefore, opt to avoid some of the ecological and physiological costs of torpor through use of shallower torpor. Further, we found no evidence that heavier bats, or bats with a higher body condition index, exhibited higher minimum or average *T*_sk_’s, which has been found in studies of little brown myotis (*Myotis lucifugus*) [9]. Comparison of our results to studies of little brown myotis should be interpreted with caution, however, as our sample sizes were small, and because we did not concurrently monitor little brown myotis in our study area.

Although we present the first published data on winter torpor patterns of Rafinesque’s big-eared bat, frequent movements during winter by this species have been reported for decades [22]. Rafinesque’s big-eared bat copulates during winter, and periodic arousals from hibernation, as well as switching among hibernacula, may play important roles in the breeding biology of this species [26], [27]. We did not document winter copulation, but a large amount of stored sperm at the base of the tail was immediately evident in all captured males, indicating that mating likely occurs throughout the hibernation season. We also found evidence that suggests big-eared bats encounter more potential mates during the winter than they encounter during other times of the year. Both males and females switched hibernacula frequently, and traveled up to 5964 m between consecutive roosts during winter months, compared to a maximum distance of 3395 m observed during three years of summer research at Mammoth Cave National Park (J. Johnson, unpublished data). Long distance movements between hibernacula were not uncommon, as we tracked 8 bats (40%; *n* = 4 males, 4 females) >2 km between consecutive hibernacula. By comparison, only 3 of 64 (5%) bats radio-tracked during the summer traveled >2 km between consecutive roosts (J. Johnson, unpubl.data). We tracked 3 female and 2 male bats >4 km between hibernacula, longer than distances reported for this species during summer research in other parts of the range [24], [40]. On several occasions bats travelled >4 km between consecutive hibernacula, only to return to the previous roost after several days.

Frequent movement among distant hibernacula may serve to maximize gene flow among populations rarely interacting during the summer. Similar movements among hibernacula, accompanied by mating, may also occur during the fall, but there are no published data of these behaviors in Rafinesque’s big-eared bat. Our finding that both sexes make these movements does not support the hypothesis of Clark [27] that Rafinesque’s big-eared bat has a resource-defense polygynous mating system. Furthermore, activity of PIT-tagged adult males was relatively high at an artificial roost from November–February, with activity of up to 47% (*n* = 17) of the PIT-tagged population on the same night in December. It is unlikely that males were engaging in territorial defense with so many prospective competitors, but it is certain that winter mating does occur at this roost, as one of the authors (S. Thomas) observed copulation among bats in the roost prior to this study.

The finding that *T*_a_ at sunset influenced probability of arousal is not surprising given that the majority of arousals occurred within an hour of sunset. We postulate that periodic arousal from torpor is under circadian control in Rafinesque’s big-eared bats, and that arousal near sunset on relatively warm nights is adaptive for winter foraging. The finding that several bats exhibited periods of thermogenesis associated with sunset, where *T*_sk_ dropped before reaching 20°C, is intriguing. We suggest that big-eared bats frequently experience a physiological state between normothermy and deep torpor, possibly to assess ambient conditions.

Studies of other cave hibernating bat species have also documented arousals during warm winter evenings, presumably providing bats with opportunities to feed and drink [41]. The brown long-eared bat (*Plecotus auritus*), frequently arouses during winter, and the ability to glean prey may allow for more effective foraging on cold evenings [41], [42]. Rafinesque’s big-eared bat is also a gleaner, and may be similarly adapted to winter foraging. An intrinsic ability to effectively feed during winter may enhance winter survivorship in gleaning species of bats by providing the energy necessary to sustain frequent arousals from torpor. Indirect evidence of winter foraging has also been found in the greater horseshoe bat (*Rhinolophus ferrumequinum*), which arouses frequently from torpor during winter, and may be capable of gleaning as noted for other *Rhinolophus* species [43], [44], [45].

As predicted, our data show Rafinesque’s big-eared bats use shorter torpor bouts during hibernation than typically reported among eastern North American cave hibernators [7], [8], [37], [46], [47]. Twente et al. [8] found that hibernacula temperature affected torpor bout duration of several species in captivity. Average and maximum torpor bout duration of little brown myotis (6.6, 26.2 d), big brown bats (*Eptesicus fuscus,* 4.1, 23.3 d), and tricolored bats (*Perimyotis subflavus*, 4.8, 34.8 d) kept at relatively high temperatures (10–11°C) were notably longer than we recorded for Rafinesque’s big-eared bats, suggesting that species differences had more influence on differences in torpor duration among species than differences in climate. We predict that other *Corynorhinus* species exhibit similar winter torpor behaviors, and encourage further research on this genus in this area.

Torpor patterns of Rafinesque’s big-eared bat are more similar to some European bats than North American species. As previously discussed, frequent winter arousals have long been observed in the brown long-eared bat [41], although torpor data from individual bats are currently lacking. Greater horseshoe bats in England used torpor bouts lasting 0.1–11.8 d (individual bats averaging 1.3–7.4 d), with torpid *T*_sk_ ranging 5–16°C (typically 10°C), and duration of normothermic periods correlated with ambient temperatures on nights >10°C [44]. Natterer’s bats (*Myotis nattereri*) hibernating in England used slightly longer torpor bouts, ranging 0.1–20.4 d (individual bats averaging 0.9–8.9 d), and with torpid *T*_sk_ centered around 10°C [48]. Interestingly, several of these studies occurred in regions with relatively mild climates similar to that in our study area.

Recent evidence suggests frequency of periodic arousals may play a role in susceptibility to mortality associated with WNS. Reeder et al. [7] found that study site and WNS infection status of bats influenced torpor bout duration. Little brown myotis unaffected with WNS exhibited the greatest average torpor bout duration (16.3 d), and remarkably, even bats which died from WNS exhibited an average torpor bout duration (7.9 d) over two times greater than the average we recorded for Rafinesque’s big-eared bats. Regional climates are also likely to influence torpor duration, however, making direct comparisons difficult. Regardless, these data suggest that little brown myotis are not adapted to survive periodic arousals at the frequency we observed for Rafinesque’s big-eared bats unless they can actively feed throughout the winter. Although we did not study torpor behaviors of little brown myotis at our study site, little brown myotis exhibiting clinical signs of WNS were documented in Kentucky concurrent with our research.

It is notable that winter torpor in Rafinesque’s big-eared bat is similar to some European bat species given the emerging evidence for the presence of *G. destructans* across Europe. Although it is not certain whether or not *G. destructans* causes mortality in European species, mass mortality has not been observed, and there is some evidence that bats groom the fungus off their bodies while normothermic [15], [49]. Currently, *G. destructans* has only been confirmed on bats of *Myotis* species in Europe. *G. destructans* has not been confirmed on *Rhinolophus* species, despite their occupation of hibernacula where the fungus is present on other species, perhaps comparable to Virginia big-eared bats in West Virginia [15], [21]. Martínková et al. [49], however, report that several photographs of *Rhinolophus hipposideros* suggest the presences of the fungus. Sign or presence of *G. destructans* has also not been documented on *Plecotus* species. We encourage researchers in Europe and North America to further investigate the comparative ecophysiology of bats with different winter torpor strategies and their susceptibility to WNS.

These data have important implications for understanding the spread of WNS in North America. Rafinesque’s big-eared bat is a rare species, and little data are available on its winter ecology. In the southern portion of the range, where Rafinesque’s big-eared bat often overwinters in hollow trees [24], it is unlikely that WNS is a major conservation concern, but populations at the northern edge of the species range rely on caves and mines for hibernacula, and infection with WNS could endanger the viability these populations [23]. We found that populations hibernating in caves in Kentucky are shallow hibernators, and their unique winter ecology may provide them with an ecological and physiological defense against the *G. destructans* fungus. We hypothesize that frequent arousals from torpor in these bats results in a more active immune system, opportunity to groom the fungus off their bodies, or opportunity to feed and drink during winter [15], [50], [51]. Additional ecological defenses may also exist, as Stihler [21] noted that Virginia big-eared bats hibernate in less humid portions of caves than species affected by WNS.

Although winter torpor behavior of Rafinesque’s big-eared bat may provide an advantage against *G. destructans*, fungal infection may still occur similar to several European species which survive colonization [15], and fungal spores may still collect on the skin and pelage. Thus, the frequent, relatively long-distance movements among cave hibernacula and abandoned buildings that we observed suggest Rafinesque’s big-eared bat may act as a vector for spread of *G. destructans*. Long-distance movements for Rafinesque’s big-eared bats are short in comparison to long-distance migrations of some *Myotis* species [52], so spread, if any, of the *G. destructans* fungus by big-eared bats is likely to be spatially limited.
